# Zero-Dimensional Hybrid Organic–Inorganic Lead Halides and Their Post-Synthesis Reversible Transformation into Three-Dimensional Perovskites

**DOI:** 10.1021/acs.inorgchem.1c00212

**Published:** 2021-03-11

**Authors:** Bas A. H. Huisman, Francisco Palazon, Henk J. Bolink

**Affiliations:** Instituto de Ciencia Molecular, Universidad de Valencia, C/ Catedrático J. Beltrán 2, Paterna 46980, Spain.

## Abstract

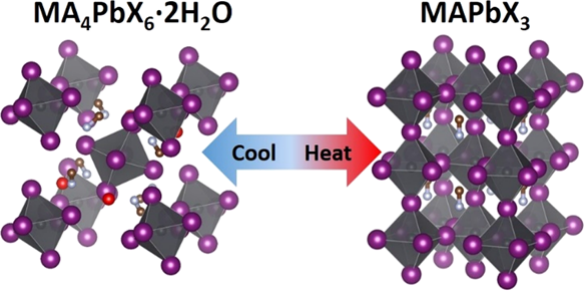

Zero-dimensional (0D) mixed-halide hybrid organic–inorganic MA_4_PbX_6_·2H_2_O (MA = CH_3_NH_3_^+^; X = Br_1 – *x*_I*_x_* with 0 < *x* < 1) has been synthesized by a solvent-free mechanochemical approach. It has been shown that this 0D phase with sharp absorption features in the near-UV is a hydrated structure, which can be reversibly transformed into the three-dimensional perovskite phase MAPbX_3_ by simple thermal annealing (dehydration) in air. This work reveals a new approach to hybrid organic–inorganic perovskites and related 0D structures, which have so far only been thoroughly studied for the inorganic Cs_4_PbX_6_ compounds.

## Abstract 2

Zero-dimensional hydrated methylammonium lead halides with narrow-band absorption in the UV are mechanochemically synthesized with different anion compositions. It is furthermore demonstrated that these structures undergo a reversible phase transformation into three-dimensional perovskites with broadband absorption throughout the visible range. These transformations occur upon simple heating in air and are shown to be linked to the desorption of water and consequent collapse of the zero-dimensional structure, contrary to what is observed on inorganic counterparts (Cs_4_PbX_6_).

## Introduction

**Figure 1:**
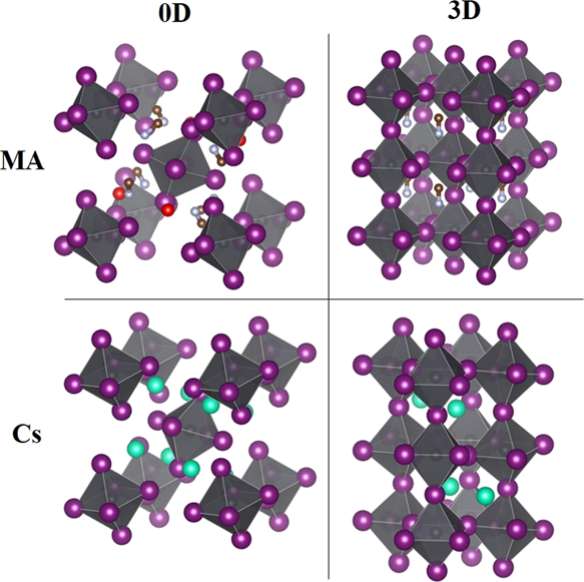
Crystal structures of MA_4_PbI_6_·2H_2_O (top left), MAPbI_3_ (top right), Cs_4_PbBr_6_ (bottom left), and CsPbBr_3_ (bottom right) drawn with VESTA software based on reference crystallographic information files retrieved from the Inorganic Crystal Structure Database (refs: 162158, 97851, and 110630) and the Crystallography Open Database (ref: 4335638). The two structures on the left can be considered as zero-dimensional based on the isolated [PbX_6_]^4+^ octahedra, while the two structures on the right can be considered three-dimensional distorted perovskites. Note that these are the most commonly reported structures for the aforementioned compounds in ambient conditions, but both MAPbI_3_ and CsPbBr_3_ convert to the so-called perfect cubic perovskite structure at high temperature.

Inorganic and hybrid organic–inorganic ternary lead halides can exist in different stoichiometries and crystal structures.^1^ APbX_3_ perovskites with A being either an alkali metal cation (e.g., Cs^+^) or a small organic cation (e.g., MA = (CH_3_NH_3_)^+^ or FA = CH(NH_2_)_2_^+^) and X a halide anion (e.g., Cl^–^, Br^–^, or I^–^) are by far the most studied class of ternary metal halides, owing to their exceptional optoelectronic properties.^2−4^ Nevertheless, other phases such as A_4_PbX_6_ or the dihydrate A_4_PbX_6_·2H_2_O have also been reported. These can be viewed as zero-dimensional analogs to the three-dimensional (3D) perovskites, based on the degree of connectivity between adjacent [PbX_6_]^4+^ octahedra^5^ (see Figure 1).

While inorganic Cs_4_PbX_6_ compounds have been extensively studied in the last 3–4 years,^6−11^ it is striking to note that only very few reports have focused on the MA- or FA-based hybrid organic–inorganic analogs.^12,13^ Yet, the existence of the dihydrate MA_4_PbI_6_·2H_2_O is known at least since 1987,^14^ and other recent articles have noted the occurrence of this phase, mostly as a degradation or side-product of the 3D perovskite counterpart.^15,16^ In fact, one of the main reasons why zero-dimensional ternary metal halides are under study is not only their intrinsic properties such as sharp absorption features in the near-UV (which could pave the way to their implementation as narrow-band UV photodetectors) but their possible interconversion into perovskites. For inorganic Cs–Pb–X compounds, an extensive literature has developed in the past few years on reversible and irreversible phase transformations under different physical and chemical stimuli.^17−21^ Only recently, these transformations have been rationalized in terms of the Cs^+^ cation substructure.^1^ The conservation of this (slightly distorted) cationic substructure is thought to be key in the interconversion of PbX_2_-rich (here APbX_3_) and PbX_2_-poor (here A_4_PbX_6_) phases. Hence, it is important to study the possibility of such interconversion with another monovalent cation, especially a small organic molecule. This is particularly important as the majority of lead halide perovskites used in optoelectronics is based on such organic cations. Furthermore, it is important to highlight that the reported hybrid organic–inorganic zero-dimensional phase is in fact a hydrated structure: MA_4_PbX_6_·2H_2_O (note the oxygen atoms from H_2_O molecules represented by red balls in Figure 1; hydrogen atoms are not shown).^14^ This added complexity also provides another possible key to the control of crystalline structures in hybrid organic–inorganic lead halides through the addition or removal of water (moisture).

Hereafter, we will show the first solvent-free mechanochemical synthesis of MA_4_PbI_6_·2H_2_O and demonstrate its reversible transformation into and from the 3D perovskite analog (MAPbI_3_) by controlled (de)hydration under thermal annealing and simple cooling down in moist air. Finally, we will demonstrate the synthesis of mixed MA_4_Pb(Br_1 – *x*_I*_x_*)_6_·2H_2_O with 0 < *x* < 1, thus expanding the possibilities of these overlooked hybrid organic–inorganic ternary metal halides, which could be implemented for instance in tunable narrow-band near-UV photodetectors.

## Results and Discussion

**Figure 2:**
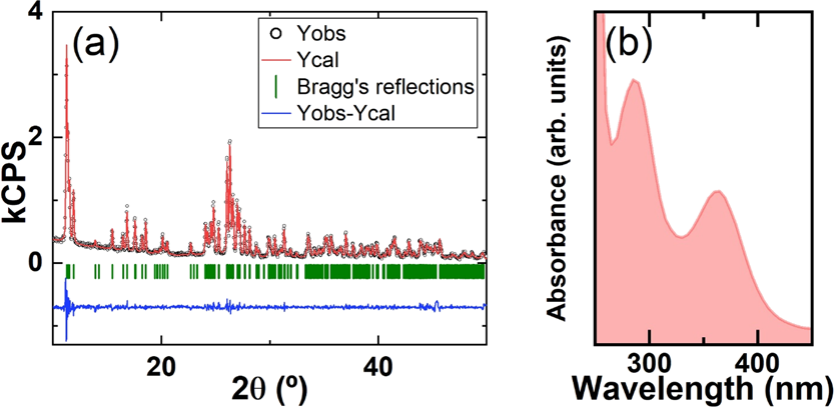
(a) XRD observed and calculated data of MA_4_PbI_6_ powder. (b) Optical absorption spectrum of a dilute dispersion of MA_4_PbI_6_·2H_2_O in a 1:3 ethanol/hexane mixture.

In order to investigate zero-dimensional hybrid organic–inorganic lead halides, MAI and PbI_2_ powders have been ball-milled in a 4:1 molar ratio in air (relative humidity around 40–60%; see the Experimental section for more details). This simple mechanochemical approach has been demonstrated to be very efficient for the synthesis of different metal halide semiconductors and phosphors.^22^ In particular, it has been implemented for the synthesis of the inorganic analogs Cs_4_PbX_6_.^23^Figure 2a shows the X-ray diffraction data of the as-obtained powders together with the calculated signal from whole-pattern deconvolution following the Le Bail fit procedure. The fit is obtained considering a P2_1_/c space group (monoclinic system) with unit cell parameters detailed in Table 1.

**Table 1:** Calculated and reference unit cell parameters of MA_4_PbI_6_·2H_2_O

space group: P2_1_/c (14)	this work	reference (ICSD:110630)^14^
a (Å)	10.469	10.421
b (Å)	11.363	11.334
c (Å)	10.701	10.668
α (°)	90	90
β (°)	91.76	91.73
γ (°)	90	90

As can be observed, the match between the observed and calculated XRD signal is very close, pointing out to the high purity of the mechanochemically synthesized zero-dimensional phase. Also, the unit cell parameters are in close agreement with the values reported by Vincent et al. from solution synthesis^14^ (see Table 1). It is important to notice that when the salt precursors are ball-milled in dry nitrogen in the same 4:1 ratio, full conversion into the desired MA_4_PbI_6_·2H_2_O phase is not achieved (see Figure S1). This highlights the importance of moisture in the formation of the zero-dimensional hybrid organic–inorganic phase, which is an important difference with the inorganic analog Cs_4_PbI_6_.

Optical absorption measurements were carried out on the as-synthesized powders dispersed in a 1:3 ethanol/hexane mixture (see the Methods section for details). Figure 2b reveals two sharp absorption peaks at 288 and 364 nm, very similar to what is observed in the absorption spectrum of Cs_4_PbI_6_.^19^ Indeed, due to the zero-dimensional structure of these compounds (see Figure 1), the optical absorption is related to the electronic configuration of isolated [PbI_6_]^4+^ octahedra. Hence, the role of the monovalent cation (MA^+^ or Cs^+^), and in this case of the water molecules, is only to preserve the structural stability. An ongoing debate on the much-more studied inorganic counterparts is centered on the possible photoluminescence (PL) from these zero-dimensional (0D) phases. While some have claimed that these do not show PL (and ascribed the observed signals to traces of 3D-phase impurities), others have attributed visible PL (at least in the case of bromide compounds) to self-trapped excitons or other intrinsic features of the 0D phase.^6,24^ We, however, could not observe any PL from these materials.

**Figure 3:**
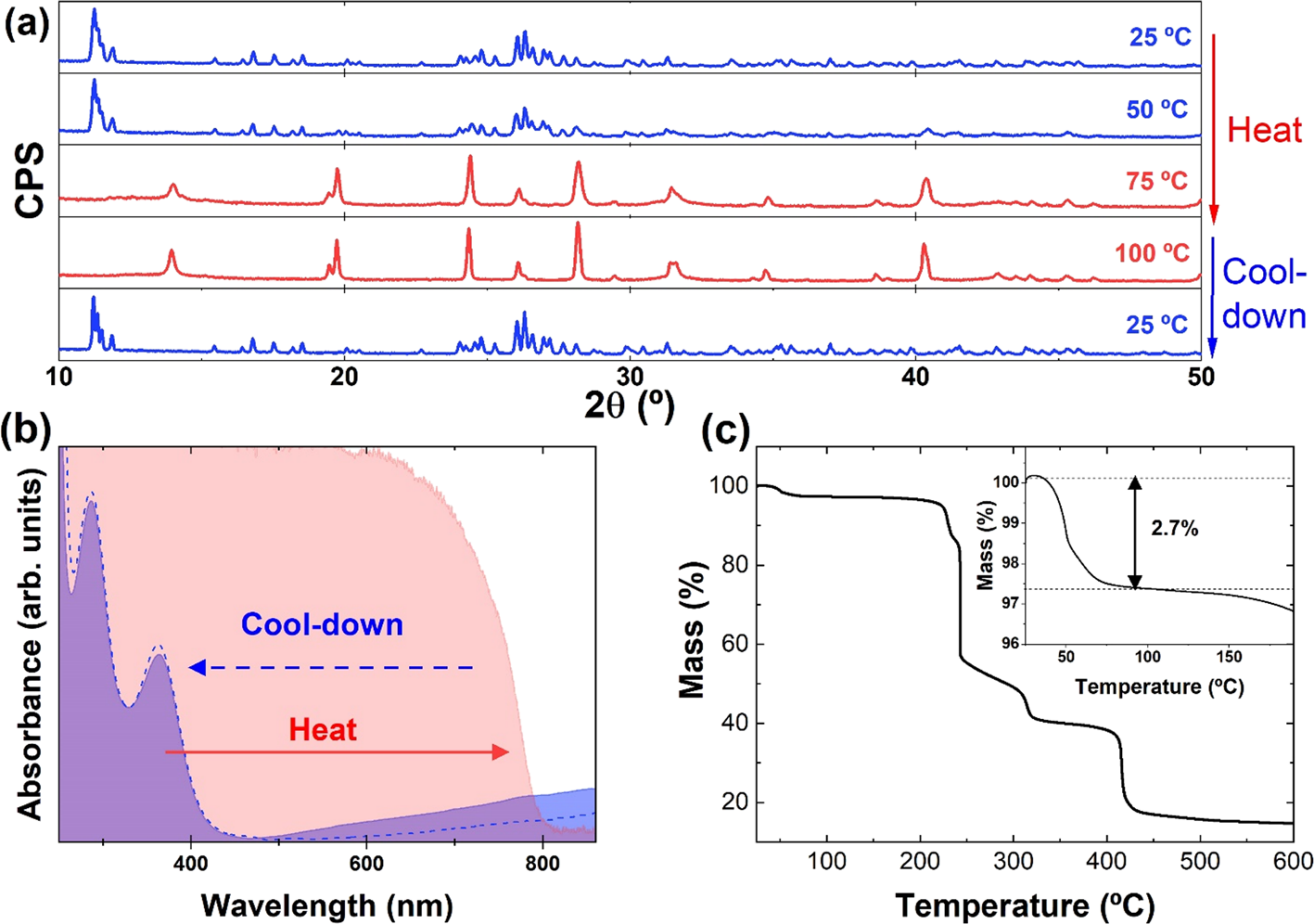
(a) Temperature-dependent X-ray diffractograms of the methylammonium lead iodide sample. (b) Optical absorption of the pristine methylammonium lead iodide sample (plain blue), as well as after thermal annealing at 100 °C (red) and upon cooling down to ambient temperature (blue dashes). (c) Thermogravimetric analysis in the 25–600 °C temperature range, with the inset focusing on the 25–175 °C range.

As previously explained, part of the interest on 0D ternary lead halides arises from their possible conversion into 3D perovskites, which are relevant for photovoltaics and other optoelectronic applications. Here, we conducted XRD of the as-synthesized MA_4_PbI_6_·2H_2_O powders while thermally annealing *in-situ* (Figure 3a).

The top diffractogram in Figure 3a, recorded at room temperature, corresponds to the 0D phase as detailed above (see Figure 2a and Table 1). When the sample is heated up to 50 °C, no significant differences are observed, indicating that this phase is stable up to this temperature. However, when thermal annealing is further conducted at 75 and 100 °C a drastic change occurs, with the disappearance of the characteristic 0D peaks and the rise of new diffraction peaks, especially at 2θ = 14.0, 19.7, 24.4, 28.2, and 40.4°. These can all be ascribed to the cubic phase of MAPbI_3_^25^ with the corresponding planes: (001), (011), (111), (002), and (022). We also note the presence of other minor peaks, as for example around 2θ = 19.4° in partial overlap with the (011) peak of MAPbI_3_. This does not seem to belong to either the 3D phase (neither cubic nor tetragonal MAPbI_3_) or the 0D one. The most likely explanation is that it belongs to crystalline CH_3_NH_3_I (MAI). Indeed, as the 0D and 3D phases are not stoichiometric, the transformation from the former to the latter necessarily involves byproducts. Furthermore, the fate of the water molecules remains unknown. It is possible that they are evaporated (note that evaporation occurs typically below the boiling point). Hence, the easiest and most straightforward reaction mechanism is the following decomposition:
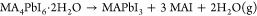
1

Nevertheless, due to the lack of a reliable crystallographic information file for MAI, we cannot guarantee that this simple reaction is the (only) one at play in the transformation observed here.

In any case, the 0D → 3D transformation is also clear from UV–visible absorption spectra (Figure 3b). Indeed, as the powders are heated up to 100 °C, a clear absorption onset forms around 800 nm (i.e., 1.55 eV) as expected for MAPbI_3_. Moreover, if the powders are left to cool down at room temperature in ambient air (relative humidity around 40–60%) for one week, both the XRD (Figure 3a, bottom diffractogram) and absorption properties of the material return to the original ones for the as-synthesized 0D phase.

To gain more detailed insights into the mechanisms involved in these transformations, we performed thermogravimetric analysis (TGA; Figure 3c) of as-synthesized MA_4_PbI_6_·2H_2_O. If we focus on the 25 °C–150 °C temperature range (highlighted in the inset in Figure 3c), we observe a mass loss of about 2.7% around 50 °C–75 °C. This loss, which corresponds to the 0D → 3D transformation observed by XRD, is consistent with the loss of two water molecules (36 g/mol) out of each MA_4_PbI_6_·2H_2_O unit (1121 g/mol). Again, this points out to the complete loss of water by evaporation at these relatively low temperatures (and not the formation of liquid water or other hydrated or solvated compounds). It is also clear that the dehydration causes the collapse of the 0D structure, which is unstable without the corresponding water molecules (as also observed when direct synthesis is attempted in dry nitrogen; Figure S1). The fact that no further weight loss is observed up to 100 °C explains that a reversed hydration in ambient air may be sufficient to recover the 0D phase, as previously observed. A further look at the TGA signal suggests that the reversibility may be preserved up to around 200 °C. However, beyond this temperature, many mass losses are observed. Indeed, the decomposition mechanism of methylammonium lead iodide can be quite complex and give rise to different species such as CH_3_NH_2_ and HI or other.^26,27^

Eventually, given the high phase purity observed on the direct mechanochemical synthesis of MA_4_PbI_6_ and the promising conversion into the 3D perovskite counterpart described so far, we decided to expand the material compositions to MA_4_PbBr_6_·2H_2_O and mixed-halide MA_4_Pb(I:Br)_6_·2H_2_O with different I/Br ratios. Indeed, mixed-halide compositions have been demonstrated to be very interesting in ternary metal halides to tune the structural and/or optoelectronic properties.

**Figure 4:**
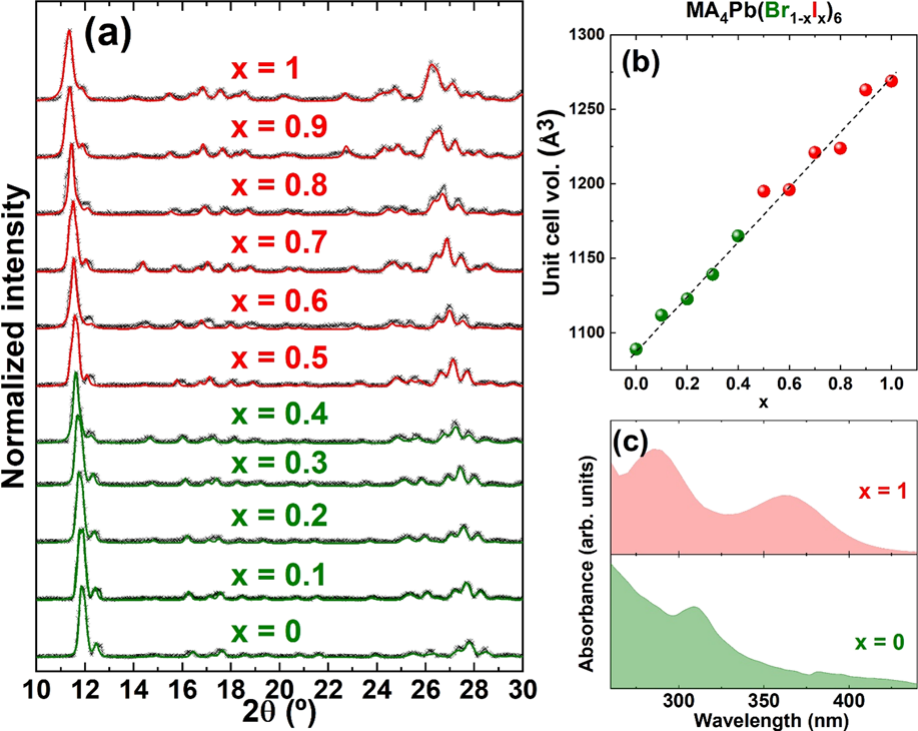
(a) XRD data of MA_4_Pb(Br_1 – *x*_I*_x_*)_6_ with 0 < *x* < 1. (b) Unit cell volume of the I–Br mixture perovskites. (c) UV–visible absorption spectra of pure MA_4_PbI_6_ and MA_4_PbBr_6_.

Figure 4a presents the XRD patterns of a series of MA_4_Pb(I*_x_*Br_1 – *x*_)_6_·2H_2_O compounds and corresponding fits.

A gradual shift toward higher diffraction angles is observed with the decreasing x value (see Figure S2 for easier visualization of the main diffraction peak shift). This is expected as the smaller anion Br^–^ replaces I^–^ in the structure, hence leading to a contraction of the unit cell. Unit cell volumes derived from Le Bail fits are represented in Figure 4b showing a linear relationship with x. Hence, we conclude that mixed MA_4_Pb(I*_x_*Br_1 – *x*_)_6_·2H_2_O 0D methylammonium lead halides follow a classical Vegaard’s law without phase transitions or miscibility gaps. This is in contrast with Cl-based compounds. In fact, we could not form Cl-analogs with this approach (see Figure S3). It may be inferred that Cl^–^ is too small to stabilize this structure. However, inorganic Cs_4_PbX_6_ compounds have been demonstrated with Cl, Br, and I.^19^ Hence, the instability of the chloride compounds may reside in the different interactions with water, as we recall that the hybrid organic–inorganic structures are hydrated. The replacement of I for Br in the crystalline structure results in a shift of the lower energy absorption peak (Figure 4c) from 364 to 310 nm, similar to what is observed on the inorganic analogs.^19^ For mixed iodide–bromide compounds, it could be expected that the absorption spectra showed features of both compositions resulting in a broader signal. This is because in such 0D structures, absorption comes from localized states in single octahedra, as explained elsewhere.^19^ In our case, we found that the absorption spectrum of the mixed I–Br compound was dominated by the absorption of PbI_6_ octahedra with no visible contribution from the bromide part (see Figure S4). We hypothesize that this may be due to a significantly higher absorption coefficient from MA_4_PbI_6_·2H_2_O compared to MA_4_PbBr_6_·2H_2_O. Nevertheless, it is worth noting that the 0D to 3D conversion upon annealing also occurs for bromide and mixed iodide–bromide compositions, and this results in tunable bandgaps in the visible range (see Figure S5).

## Methods

### Materials

Methylammonium iodide (MAI, >99.5%), methylammonium bromide (MABr, >99.5%), and lead(II) iodide (PbI_2_, ≥ 99.999%) were purchased from Lumtec. Lead(II) bromide (PbBr_2_, ≥ 98%) was purchased from TCI. All chemicals were stored in a nitrogen-filled glovebox and used as received without further purification.

### **Mechanochemical synthesis of** MA_4_Pb(Br_1 – *x*_I*_x_*)_6_·2H_2_O **powders**

MAI:MABr:PbI_2:_PbBr_2_ powders (X = Cl, Br, or I) were weighed inside a nitrogen-filled glovebox. Then, approximately 2 g of the mixed precursor powders was introduced and closed inside 10 mL zirconia ball-mill jars with two zirconia beads of 10 mm in diameter under an ambient atmosphere to introduce moisture. Then, ball-milling was performed with a MM-400 straight ball-mill from Retsch, at a frequency of 30 Hz for 1 h.

### XRD characterization

X-ray diffraction was measured with a Panalytical Empyrean diffractometer equipped with a CuKα anode operated at 45 kV and 40 mA and a Pixel 1D detector in scanning line mode. Single scans were acquired in the 2θ = 10 to 50° range in Bragg–Brentano geometry in air. The annealing of the powder was performed in-situ with a custom-made heating platform. Heating was performed at approximately 7 °C/min, and the temperature was left constant for 5 min at each step before starting the data acquisition. Data analysis, in particular Le Bail whole-pattern fits, was performed with Fullprof software.

### Optical characterization

For optical absorbance measurements, powders were dispersed in an ethanol/hexane (1:3) solution. Absorbance spectra were then collected with a PerkinElmer UV/visible spectrometer, applying a background correction (blank) for the neat solvent mixture.

### Thermogravimetric analysis (TGA)

The TGA measurements were performed with a TGA550 from TA instruments and a temperature step size of 20.0 °C/min.

## Conclusions

In summary, 0D MA_4_Pb(Br_1 – *x*_I*_x_*)_6_·2H_2_O powders (0 < *x* < 1) with sharp and tunable absorption features in the near UV have been successfully synthesized by a simple mechanochemical approach. Structural characterization reveals the high-purity and good halide mixing of all compounds. Furthermore, thermal annealing in air at moderate temperatures (around 75 °C) triggers a drastic transformation from the 0D phase into the 3D perovskite analog, with strong absorption throughout the whole visible range. This transformation is reversible by simply cooling down the sample in air. These results, especially on the reversible phase transformations, are rationalized by a (de)hydration mechanism. Indeed, contrary to what is reported for the inorganic Cs_4_PbX_6_ counterparts, water is an essential part of the dihydrate hybrid organic–inorganic MA_4_PbX_6_·2H_2_O compounds; in other words, nonhydrated MA_4_PbX_6_ does not appear to be thermodynamically stable. Our results pave the way to a better understanding of the phase transformations of ternary metal halides, which have so far only been extensively studied for Cs-based inorganic compounds.

## Supporting Information Available

X-ray diffractograms and UV–visible absorption spectra (PDF)
