# Supplementary material for: Zero-Dimensional Hybrid Organic–Inorganic Lead Halides and Their Post-Synthesis Reversible Transformation into Three-Dimensional Perovskites
Source: Inorg Chem. 2021 Mar 11;60(7):5212–6. doi: 10.1021/acs.inorgchem.1c00212 (PMC9682476; doi:10.1021/acs.inorgchem.1c00212)
Supplement: Supplementary file 1 — ic1c00212_si_001.pdf [file ic1c00212_si_001.pdf]

Supporting Information for:

Zero-dimensional hybrid organic-inorganic lead halides and their post-synthesis reversible transformation into 3D perovskites

Bas A. H. Huisman, Francisco Palazon,* and Henk J. Bolink

Instituto de Ciencia Molecular, Universidad de Valencia, C/ Catedrático J. Beltrán 2, 46980, Paterna, Spain. E-mail: francisco.palazon@uv.es

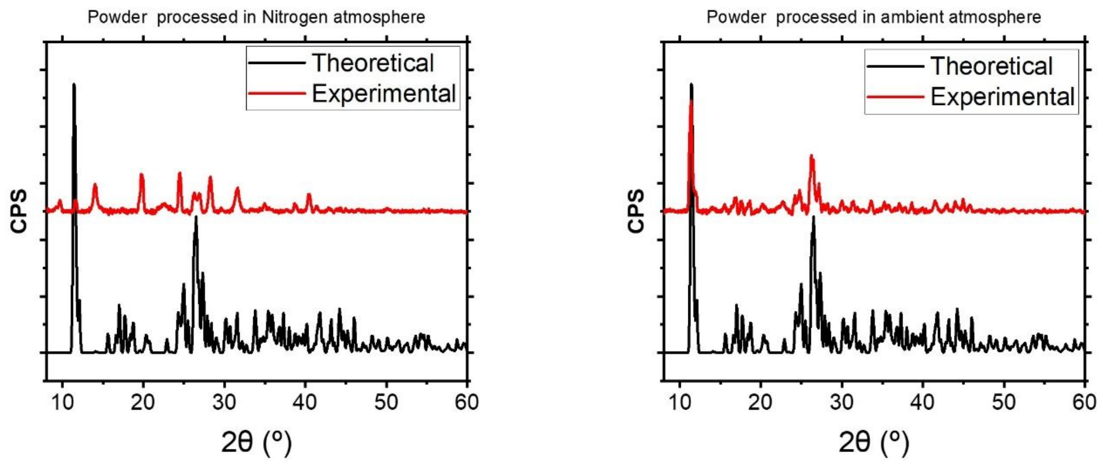

Figure S 1. X-ray diffraction patterns of ball-milled MAI:PbI_2 in 4:1 ratio in nitrogen (left panel, red) and air (right panel, red), compared to the theoretical pattern of MA4PbI6·$2H_2O$ (black). Only when ball-milling is performed in air the experimental pattern shows a good match with the expected 0D phase.

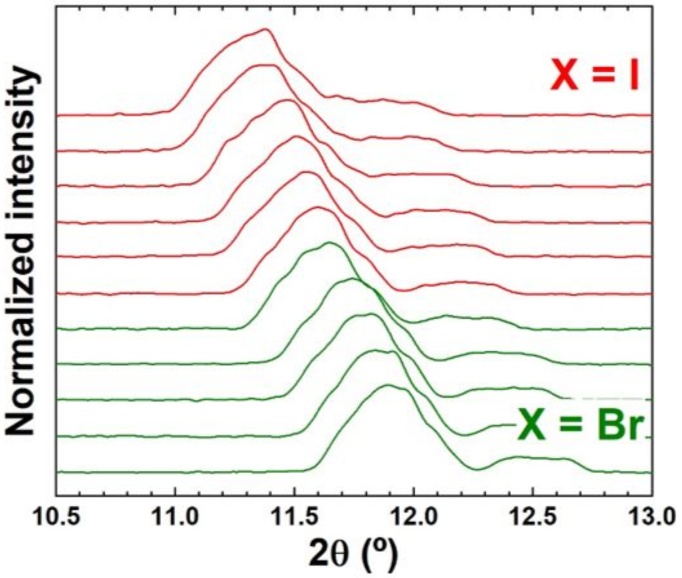

Figure S 2. X-ray diffractograms of $MA_4Pb(Br_{1-x}I_x)_6{\cdot}2H_2O$. Top diffractogram corresponds to x=1 and bottom one to x=0. This is a closer view on the low-angle part of the data presented in the main text (Figure 4b) for a better visualization of the diffraction signal shift corresponding to the unit cell expansion with the gradual replacement of bromide with iodide anions.

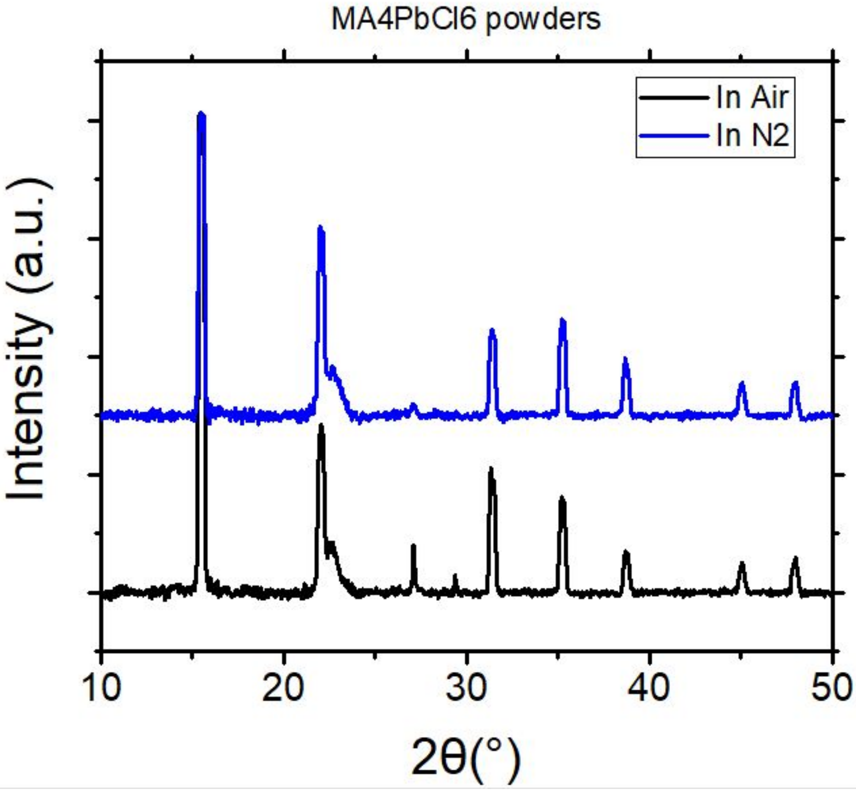

Figure S 3. X-ray diffraction pattern of ball-milled $MACl{:}PbCl_2$ powders in 4:1 molar ratio in air and nitrogen. In both cases the observed phase corresponds to $MAPbCl_3$.

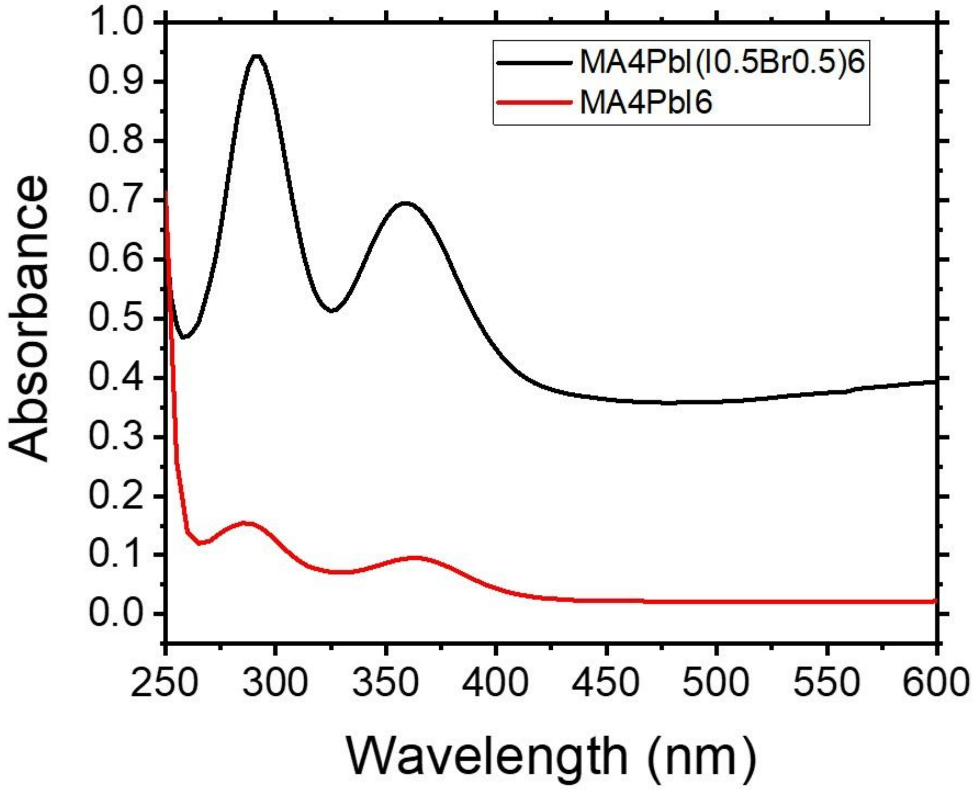

Figure S 4. UV absorption spectra of $MA_4Pb(Br_{0.5}I_{0.5})_6{\cdot}2H_2O$ and $MA_4PbI_6{\cdot}2H_2O$ showing nearly identical features. This is not the case for 3D compounds after annealing (see Figure S5).

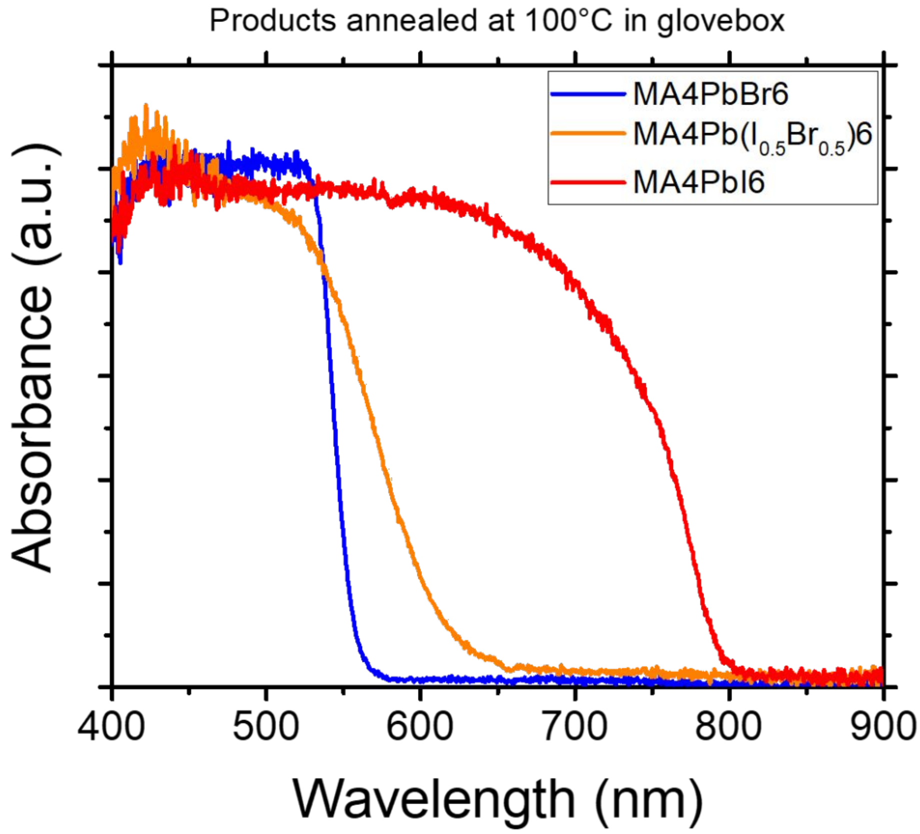

Figure S 5. Absorption spectra of annealed powders with different compositions, showing the formation of three-dimensional perovskties MAPbBr3 (blue), MAPbI3 (red), and mixed MAPb(I:Br)3 (orange).
